# Oxidative stress and impaired oligodendrocyte precursor cell differentiation in neurological disorders

**DOI:** 10.1007/s00018-021-03802-0

**Published:** 2021-03-10

**Authors:** Jan Spaas, Lieve van Veggel, Melissa Schepers, Assia Tiane, Jack van Horssen, David M. Wilson, Pablo R. Moya, Elisabeth Piccart, Niels Hellings, Bert O. Eijnde, Wim Derave, Rudy Schreiber, Tim Vanmierlo

**Affiliations:** 1 University MS Center (UMSC), Hasselt-Pelt, Belgium; 2 BIOMED Biomedical Research Institute, Faculty of Medicine and Life Sciences, Hasselt University, Hasselt, Belgium; 3 Department of Movement and Sports Sciences, Faculty of Medicine and Health Sciences, Ghent University, Ghent, Belgium; 4 Department Psychiatry and Neuropsychology, Division of Translational Neuroscience, European Graduate School of Neuroscience, School for Mental Health and Neuroscience, Maastricht University, Maastricht, The Netherlands; 5 Department of Molecular Cell Biology and Immunology, Amsterdam Neuroscience, MS Center Amsterdam, Amsterdam University Medical Center, Location VUmc, Amsterdam, The Netherlands; 6 Facultad de Ciencias, Instituto de Fisiología, Centro Interdisciplinario de Neurociencia de Valparaíso (CINV), Universidad de Valparaíso, Valparaíso, Chile; 7 Faculty of Medicine and Life Sciences, SMRC-Sportsmedical Research Center, BIOMED Biomedical Research Institute, Hasselt University, Diepenbeek, Belgium

**Keywords:** Oligodendrocyte precursor cell, Oxidative stress, Carbonyl stress, Neurodegeneration, Myelination

## Abstract

Oligodendrocyte precursor cells (OPCs) account for 5% of the resident parenchymal central nervous system glial cells. OPCs are not only a back-up for the loss of oligodendrocytes that occurs due to brain injury or inflammation-induced demyelination (remyelination) but are also pivotal in plastic processes such as learning and memory (adaptive myelination). OPC differentiation into mature myelinating oligodendrocytes is controlled by a complex transcriptional network and depends on high metabolic and mitochondrial demand. Mounting evidence shows that OPC dysfunction, culminating in the lack of OPC differentiation, mediates the progression of neurodegenerative disorders such as multiple sclerosis, Alzheimer’s disease and Parkinson’s disease. Importantly, neurodegeneration is characterised by oxidative and carbonyl stress, which may primarily affect OPC plasticity due to the high metabolic demand and a limited antioxidant capacity associated with this cell type. The underlying mechanisms of how oxidative/carbonyl stress disrupt OPC differentiation remain enigmatic and a focus of current research efforts. This review proposes a role for oxidative/carbonyl stress in interfering with the transcriptional and metabolic changes required for OPC differentiation. In particular, oligodendrocyte (epi)genetics, cellular defence and repair responses, mitochondrial signalling and respiration, and lipid metabolism represent key mechanisms how oxidative/carbonyl stress may hamper OPC differentiation in neurodegenerative disorders. Understanding how oxidative/carbonyl stress impacts OPC function may pave the way for future OPC-targeted treatment strategies in neurodegenerative disorders.

## OPCs in health and disease

Upon differentiation-inducing stimuli, oligodendrocyte precursor cells (OPCs) provide the source of newly born oligodendrocytes for the myelination of neuronal axons in the central nervous system (CNS). OPCs first arise from neural stem cells during the embryonic developmental stage and persist into adulthood, constituting a significant portion (~ 5–10% of glial cells) of the adult CNS [1–4]. OPCs remain by far the most proliferative cell type in the CNS [5, 6] and contribute to the maintenance of myelination via low-rate oligodendrocyte turnover [7–9]. OPC differentiation in the adult CNS also drives ‘adaptive myelination’ during learning and memory. Indeed, myelin plasticity is increasingly being acknowledged as vital, alongside the more generally known synaptic plasticity [10]. Several studies have highlighted that newly formed myelin is essential for implicit motor skill learning [11], explicit learning, and remote (but not recent) memory consolidation and/or recall [2, 8, 12–14]. This evolving position opposes the classic view of OPCs and oligodendrocytes as being passive static insulators around neuronal axons, instead portraying them as dynamic cells that fine-tune neuronal networks and functionally affect behaviour, cognition and neurophysiology [15]. Moreover, OPCs actively survey their environment and quickly initiate repair processes following demyelination. During a process called remyelination, OPCs migrate towards the lesion site, differentiate into mature oligodendrocytes and enwrap surviving axons [16, 17]. Remyelination has been observed in a variety of traumatic and non-traumatic CNS disorders, aiming to protect denuded axons from degeneration and restore normal neuronal conduction [16, 18].

When compromised, however, impaired OPC function and (re)myelination appear to play a major role in a variety of neurodegenerative disorders [19]. Inflammation, excitotoxicity, oxidative stress, and protein aggregation are some of the major causes of oligodendrocyte pathology in multiple sclerosis (MS), Alzheimer’s disease (AD) or Parkinson’s disease (PD), as well as neuropsychiatric disorders such as schizophrenia and major depressive disorder (MDD) [19–25]. Progressive oligodendrocyte abnormalities and eventually oligodendrocyte loss strongly correlate with cognitive decline in the normal ageing brain, AD and MS [26]. Upon oligodendrocyte loss, OPC differentiation is paramount to generate new oligodendrocytes. However, a lack of OPC differentiation impedes CNS (re)myelination, a process that may be more abundant and accurate when performed by newly generated compared to surviving oligodendrocytes [27]. Even in chronically demyelinated MS lesions, the OPC pool is not depleted [21, 28]. Yet, remyelination is limited and mistargeted [27, 29], and it was estimated that surviving (but not newly generated) oligodendrocytes were the main contributors to remyelination in MS shadow plaques [30]. In schizophrenia and PD, an increase in the OPC population has even been observed in the prefrontal cortex and cerebellum, respectively [19, 31].

The reduced differentiation and (re)myelination capacity of aged OPCs appears to be further compromised in neurodegenerative disorders [32–35]. In schizophrenia, various signalling pathways are impaired, with cell division pathways upregulated (e.g. PDGF signalling) but differentiation markers downregulated (e.g. OLIG2) [19]. Disruption of brain connectivity in adolescents with schizophrenia has been linked to impaired OPC differentiation and prefrontal cortex myelination [36]. Similarly, dysregulation of the gene expression network in OPCs is strongly associated with AD [21, 37]. A recent study analysed RNA expression profiles of autopsy brain tissue from dementia patients and revealed disturbed signalling pathways involved in OPC differentiation, oligodendrocyte development, gliogenesis, myelination and axon ensheathment [38]. Disruption of signalling pathways involved in differentiation and migration of OPCs, such as PDGF-2A and FGF-2, is also observed in MS, eventually leading to a differentiation block in the more chronic stage [39]. In addition, OPC differentiation is inhibited by extracellular myelin debris derived from damaged oligodendrocytes [40]. Even in amyotrophic lateral sclerosis (ALS), comparable fate restriction of OPCs is observed, limiting repair and consequently resulting in loss of oligodendrocyte support in the brain [21, 41].

In summary, the evidence is emerging that OPC abnormalities are an important pathological hallmark in the development and/or progression of several neurodegenerative (and psychiatric) disorders. Several questions, however, remain: what are the mechanisms that give rise to OPC dysfunction? How (much) does this dysfunction affect (re)myelination and ultimately the neurophysiological and functional outcomes? At what disease stage and in what cellular environments can OPC dysfunction be effectively targeted therapeutically? In the present review, we focus on the effect of elevated oxidative stress, a common physiological phenomenon observed in ageing, neuroinflammation and neurodegeneration, on OPC differentiation. We begin by describing the transcriptional and metabolic changes required for oligodendrocyte development. Next, the physiology and sources of oxidative stress are discussed, followed by the inherent vulnerability of OPCs to oxidative stress and the effect of oxidative stress on OPC differentiation. We then discuss (epi)genetic and metabolic mechanisms that may be affected by oxidative stress in OPCs and interfere with cell differentiation/myelination. Finally, we highlight potential therapeutic approaches to tackle oxidative stress-induced OPC dysfunction and maintain myelin plasticity in neurodegenerative disease.

**Fig. 1:**
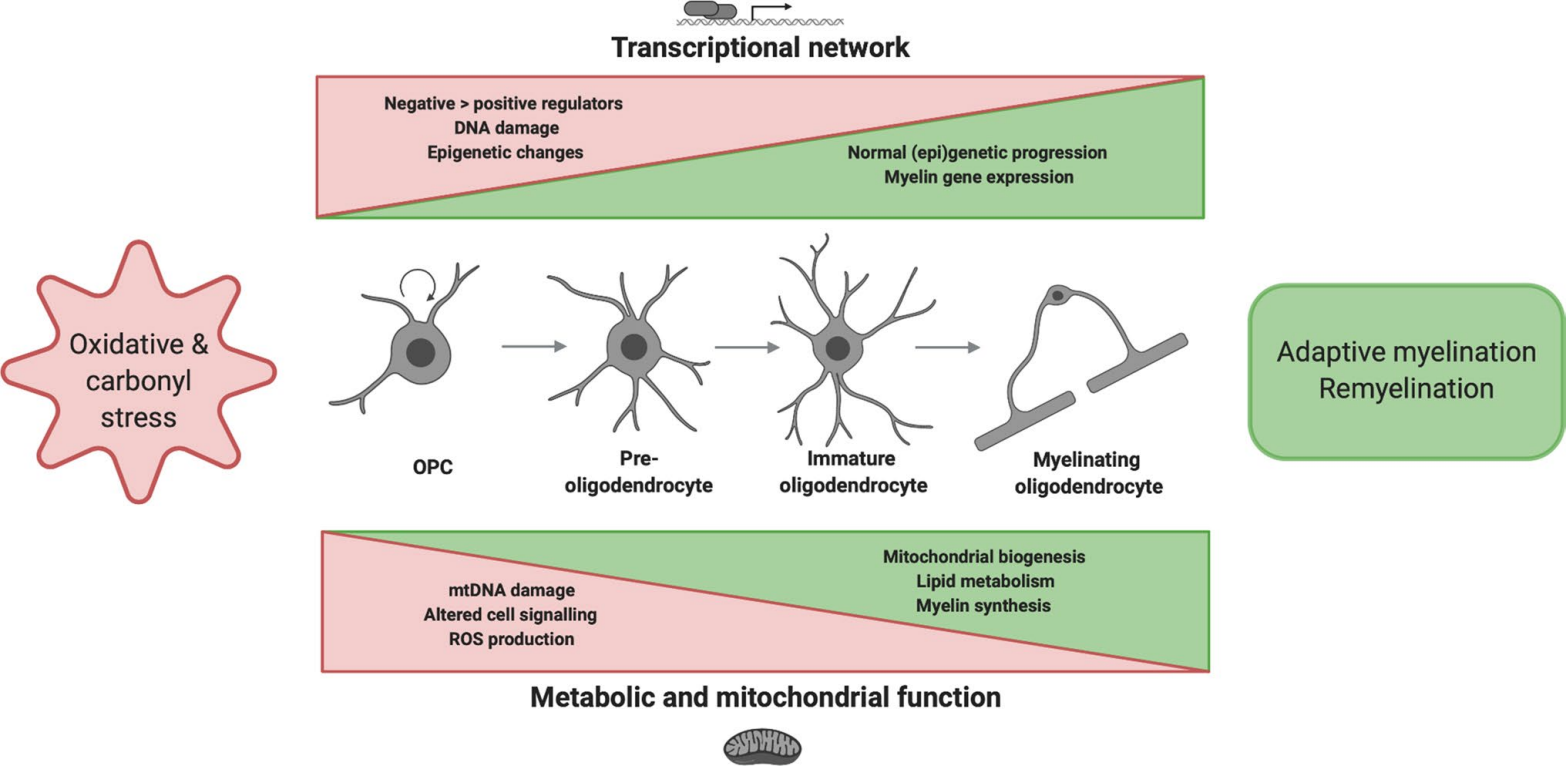
Outline of the review describing oxidative and carbonyl stress during transcriptional and metabolic changes associated with OPC differentiation. OPC differentiation occurs in at least four different stages characterised by increasing morphological complexity. By altering normal transcriptional and metabolic adaptations required for differentiation, oxidative/carbonyl stress may hamper oligodendrocyte development and consequently (re)myelination. *OPC* oligodendrocyte precursor cell; *ROS* reactive oxygen species. Figure created in BioRender

A graphical abstract-style summary of the review topic can be found in Fig. 1.

## From OPC to oligodendrocyte

Normal oligodendrocyte development and myelination are controlled by a complex transcriptional network and require a substantial metabolic demand.

### Transcriptional network for OPC differentiation

Both neonatal and adult oligodendrogenesis comprises at least four distinct stages (Fig. 1) based on cell morphology and expression of specific markers. Due to tightly controlled changes in the transcriptional network, small bipolar A2B5^+^/NG2^+^/PDGFαR^+^ OPCs are able to transform into branched axon wrapping GalC^+^/MBP^+^/PLP^+^ oligodendrocytes [42]. Advanced gene knockout studies, single-cell sequencing and bioinformatics analyses have yielded important insights into positive and negative regulators of oligodendrocyte lineage development. The most well-established positive regulators are the OLIG1, OLIG2 and SOX10 transcription factors that are expressed throughout all developmental stages [43, 44]. Other transcription factors that operate as positive differentiation regulators are expressed predominantly in the early stage (e.g. ASCL1), the late stage (e.g. NKX6.2) or biphasically (e.g. NKX2.2) [45]. Terminal oligodendrocyte differentiation requires activation of the myelination program, a process where myelin regulatory factor (MYRF) plays a pivotal role [46]. According to the ‘de-repression’ model of oligodendrocyte development, differentiation-enhancing transcription factors are initially countered by several negative regulators including ID2, ID4, HES5, SOX5, SOX6 and TCF4 [47–49]. Interestingly, reciprocal interactions between NKX2.2, OLIG2 and SOX10 have been described [50], as well as between MYRF and SOX10 [51, 52]. Hence, oligodendrocyte development reflects a complex interplay between many spatiotemporally orchestrated signals, rather than a simple positive/negative net sum.

Maintaining the transcriptomic homeostasis is a significant challenge for developing oligodendrocytes, influenced by divergent intra- and extracellular cues that act to either inhibit or facilitate OPC differentiation (e.g. growth factors) [48]. Increasing evidence also supports that epigenetic modulation of the transcriptional network—including histone modifications, DNA methylation and microRNAs (miRNAs)—governs oligodendrocyte development [53, 54]. These epigenetic mechanisms can control the activation and repression of differentiation signals in OPCs; when dysregulated, normal oligodendrocyte development is blocked.

### Metabolic and mitochondrial adaptations during differentiation

Oligodendrocytes can accommodate up to 40 myelin segments and maintain membrane extensions up to 100 × the weight of its cell body [3, 55], with immature oligodendrocytes undergoing as much as 6,500-fold increases in membrane area [56, 57]. The myelin sheath is a lipid-rich membrane built from phospholipids (~ 40%), glycolipids (~ 20%) and cholesterol (~ 40%, which is 80% of the total brain cholesterol pool) [58–60], and serves as an insulating layer around nerves that transmits electrical signals within the CNS.

Driven by (epi)genetic regulation at the nuclear and mitochondrial DNA (nDNA, mtDNA) levels, OPC differentiation and myelination go hand in hand with several metabolic adaptations. In vitro rodent and human oligodendrocyte differentiation induced many transcripts related to mitochondrial biogenesis, electron transport chain (ETC), ATP synthesis, fatty acid oxidation and cholesterol biosynthesis [61]. Moreover, the total mtDNA content increased relative to nDNA up to fourfold. Recent quantitative proteomic analysis confirmed the upregulation of multiple metabolic processes related to lipid synthesis, myelination, and cytoskeletal organisation [62]. Nevertheless, cellular metabolism, mitochondrial respiration, and cell signalling pathways in (developing) oligodendrocytes remain understudied compared to neurons. Rinholm et al. were the first to characterise mitochondrial location, morphology and movement within oligodendrocytes [63]. Mitochondria were detected in cell somata, primary processes and cytoplasmic channels of the myelin sheath, with lower mitochondrial density in the myelin sheath compared to the primary processes. Mitochondria were able to move in and out of the myelin sheaths and primary processes, albeit with lower mobility and slower speed than in neurons. A similar examination of mitochondria in OPCs or developing oligodendrocytes is lacking to date; however, an increase in the number of mitochondria in rat oligodendrocyte processes was observed following differentiation in vitro [64].

In oligodendrocytes, mitochondria are not only needed for cellular energy provision (ATP synthesis by oxidative phosphorylation), but also for lipid biosynthesis to produce myelin [58, 65]. In fact, based on their morphological properties (i.e. short length, few cristae), it has been suggested that oligodendrocyte mitochondria synthesise relatively little ATP [63]. Instead, acetyl coenzyme A (acetyl-CoA) produced in the mitochondrial matrix can be transported out of the mitochondria as citrate [61]. Acetyl-CoA is an important substrate in fatty acid or cholesterol synthesis that are central to myelin sheath formation via conversion by acetyl-CoA carboxylase (ACC) and fatty acid synthase (FASN) or 3-hydroxy-3-methylglutaryl-CoA synthase 1 (HMGCS1) and HMG-CoA reductase (HMGCR), respectively. Although acetyl-CoA is a key molecule found at the crossroads of cellular lipid biosynthesis and energy generation in oligodendrocytes, the relative proportion of acetyl-CoA derived from glucose/pyruvate, lactate or β-oxidation for these processes remains unclear [58, 66, 67]. Mitochondrial β-oxidation is assisted by peroxisomal β-oxidation that generates acetyl-CoA via breakdown of (very) long-chain fatty acids (VLCFA) [68, 69].

A healthy metabolic function is indispensable for oligodendrocytes during all stages of differentiation. The late developmental stages require extensive biosynthesis and maintenance of myelin membranes. Yet, higher in vitro ATP production and oxygen consumption rates per µg protein have been reported in OPCs compared to oligodendrocytes, highlighting that OPC differentiation is an energy-demanding process [70]. The metabolic burden of myelinating glia is tightly coupled to axonal metabolism. Once oligodendrocytes have ensheathed neuronal axons they can shuttle lactate/pyruvate via the MCT1 monocarboxylate transporter [59, 71, 72]. In addition, it was recently demonstrated that axons with thinner myelin sheaths had larger axonal mitochondria, further underscoring the importance of oligodendrocytes for neuronal health [73].

## Oxidative and carbonyl stress in OPC differentiation

### Physiology and sources of oxidative/nitrosative and carbonyl stress in OPCs

**Fig. 2:**
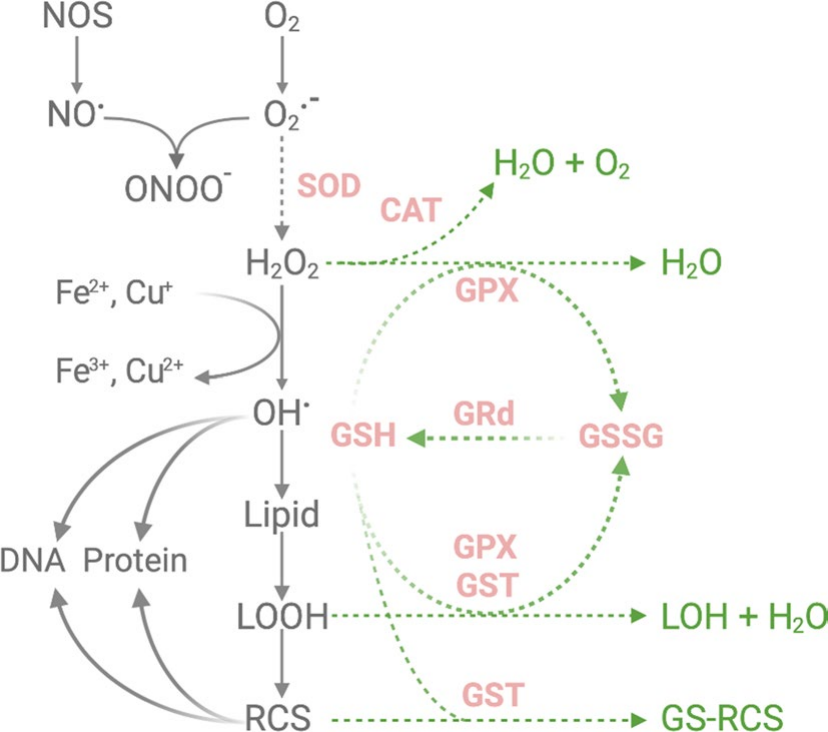
ROS generation and antioxidant defence in OPCs. The primary production and conversion pathways of ROS/RCS are shown on the left. The green arrows illustrate major antioxidant and detoxifying pathways. Enzymes and molecules with a known reduction in activity and/or expression in OPCs compared to mature oligodendrocytes and/or other CNS cell types are depicted in pink and with dotted lines*.* *CAT* catalase, *Cu* Copper, *Fe* Iron, *GPX* glutathione peroxidase, *GRd* glutathione reductase, *GSH* glutathione, *GSSG* glutathione disulphide, *GST* glutathione S-transferase, *H*_*2*_*O*_*2*_ hydrogen peroxide, *LOH*: lipid alcohol, *LOOH* lipid hydroperoxides, *NOS* nitric oxide synthase, *NO*^**·**^ nitric oxide, *O*_*2*_ oxygen, *O*_*2*_^*•−*^ superoxide, *ONOO*^*−*^ peroxynitrite, *OH*^**·**^ hydroxyl radical, *RCS* reactive carbonyl species, *SOD* superoxide dismutase. Figure created in BioRender

Oxidative stress results from a failure to control cellular pro- *vs.* anti-oxidant levels. The most well-known pro-oxidants are reactive oxygen and nitrogen species (ROS and RNS, respectively), a group of small molecules encompassing both free radical and non-radical derivatives of oxygen and nitrogen. Free radicals such as superoxide (O_2_^**·**−^) and nitric oxide (NO^**·**^) are atoms or molecules that possess at least one unpaired electron on the valence shell and are extremely reactive with short half-lives. The generally more stable non-radical species (e.g. hydrogen peroxide, H_2_O_2_) can exert similar biological effects and can emerge from or be converted to radical species in the presence of transition metals (Fig. 2).

OPCs are exposed to ROS generated intra- and extracellularly. Intracellularly, approximately 0.2–2% of electrons that pass through the electron transport chain (ETC) leak and interact with oxygen to form O_2_^**·**−^ that is released into the mitochondrial matrix (complex I) or the intermembrane space (complex III) [74–76]. Under conditions of high energy demand or mitochondrial dysfunction, increased amounts of ROS are produced. In CNS disorders, mitochondrial dysfunction has mainly been described in neurons [77, 78] and even in relation to myelin thickness [73], but less is known about oligodendrocytes or OPCs. Studies on non-mitochondrial intracellular ROS/RNS sources in OPCs and oligodendrocytes are also limited, but include inducible nitric oxide synthase (iNOS), neuronal NOS (nNOS) [79–81] and NADPH oxidase (NOX) enzymes [82, 83], as well as peroxisomal lipid metabolism [84]. In vitro studies have shown mitochondrial or enzymatic ROS production in OPCs/oligodendrocytes upon exposure to a variety of stimuli, such as proinflammatory cytokines [85, 86], VLCFA [84] and oxygen–glucose deprivation [87–89]. OPCs express ionotropic glutamate receptors (AMPARs, KARs, NMDARs) and metabotropic glutamate receptors (mGluRs), and can receive synaptic input from over a hundred neurons; however, these synapses are lost as OPCs mature into oligodendrocytes [90, 91]. Sustained activation of either AMPA or kainate receptors in oligodendrocytes causes a harmful increase of cytosolic Ca^2+^, which results in mitochondrial depolarization, an increase in ROS and, consequently, cell death [87, 92–94]. However, it should be noted that under physiological conditions, AMPA receptors are activated for mere milliseconds. Activation of NMDA receptors stimulates protein kinase C (PKC), which in its turn activates NOX2 and generates ROS [95]. Group I mGluRs, on the other hand, can attenuate the excitotoxicity and ROS accumulation that results from overactivation of ionic glutamate receptors [87, 96]. Clearly, mitochondria play a key role in these processes, by acting as a buffer organelle for Ca^2+^ and Fe-ions [97–99] that is directly linked to redox metabolism and cell signalling. Hereby, mitochondrial redox state is a powerful regulator of gene transcription, protein translation machinery and cell growth, but also apoptosis (e.g. via opening of mitochondrial permeability transition pore) [100, 101]. Interestingly, greater mitochondrial activity, resulting in higher ROS generation and thereby the risk of oxidative injury, has been observed in neurological disorders such as MS [101–103]. So far, this has predominantly been reported in axons as a result of demyelination, but it would be interesting to also assess this in OPCs and oligodendrocytes themselves [101, 102, 104, 105].

Besides the intracellular threats, extracellular ROS released by pro-inflammatory cells, such as microglia and macrophages, can attack neighbouring OPCs/oligodendrocytes [106, 107]. It is important to note that the effect of ROS in circulating or infiltrating immune cells depends on the site of production, especially in case of autoimmunity where ROS induce Treg activation while limiting T cell proliferation and cytokine production [108–111], resulting in dampened disease activity. Similarly, increased ROS generation by peripheral blood monocytes via *NOX3* is associated with better response to dimethyl fumarate in MS patients. Within the CNS parenchyma, however, ROS production is frequently linked to tissue injury [108, 109]. Acute inflammatory activity of microglia and infiltrated macrophages causes an oxidative burst, but chronic low-grade microglia/astrocyte activation is also a frequent characteristic of neurological disease. For example, high expression of iNOS and NOX complexes 1–5 (mainly NOX2) are the main drivers of oxidative injury in active MS lesions, the prototype of CNS inflammation [112, 113]. Adjacent normal-appearing white matter (NAWM) [114, 115] and macroscopically-invisible preactive lesions [116] also display microglial inflammation and NOX expression, indicating widespread ROS production in MS brain tissue. MDD [117], schizophrenia [118, 119], AD [120, 121], PD [122, 123] and stroke [124] are all characterised by abundant microglial activation and inflammation-mediated oxidative stress. Interestingly, when pro-oxidant levels are elevated in the CNS (by knockout of the antioxidant transcription factor nuclear factor erythroid 2-related factor 2, Nrf2), increased oligodendrocyte damage and demyelination is observed [125]. Alternatively, upregulation of the antioxidant defence system in OPCs rescues them from oxidative stress-induced damage [126–128].

ROS, RNS, and their reaction products (e.g. peroxynitrite, ONOO^−^) attack all major classes of biomolecules including lipids, DNA, RNA and proteins. The CNS is prone to lipid peroxidation due to abundant membrane poly-unsaturated fatty acids (PUFAs), as well as high oxygen consumption and iron levels [129]. The process of lipid peroxidation is initiated by hydrogen abstraction, creating a carbon-centred radical (L^•^). The peroxyl radical (LOO^•^) that is formed following rearrangement and O_2_ addition is then capable of starting a chain reaction of repetitive hydrogen abstraction from other PUFAs (propagation). Common end products of these reactions, the lipid hydroperoxides (LOOH), are broken down to cytotoxic aldehydes (Fig. 2). Different structural categories of aldehydes include (1) α,β-unsaturated aldehydes, such as 4-hydroxynonenal (HNE) and acrolein, (2) keto aldehydes, such as methylglyoxal, (3) dialdehydes, such as malondialdehyde and glyoxal [130–135]. These lipid-derived aldehydes (or lipid-derived reactive carbonyl species [RCS]) rapidly form adducts with phospholipids, DNA, and His, Cys, Lys and Arg residues on proteins. Although many ROS have a half-life below one second, RCS can last hours or even days and diffuse out of the cell, inciting cell damage and a variety of signalling effects [136–139].

### Endogenous defence mechanisms and OPC vulnerability

The content and activity of enzymatic and non-enzymatic antioxidant defence strategies are limited in oligodendrocytes and in particular in OPCs (Fig. 2). Superoxide dismutase (SOD) is of primary importance for the enzymatic conversion of O_2_^**·**−^ to H_2_O_2_. Three isoforms of SOD, copper/zinc-SOD (SOD1) in the cytosol, manganese-SOD (SOD2) in mitochondria, and extracellular SOD (SOD3), have been identified so far [140]. In OPCs, SOD2 protein levels and activity are up to fourfold lower compared to mature oligodendrocytes [128, 141]. In MDD, a further reduction of SOD1 and SOD2 expression in the oligodendrocyte lineage is reported, suggesting even higher vulnerability in a diseased state [142]. H_2_O_2_ is converted by enzymatic antioxidants such as catalase (CAT) and glutathione peroxidases (GPXs). CAT does not play a crucial role at low levels of H_2_O_2_ but becomes more important when these levels increase [143]. GPXs reduce H_2_O_2_ by using glutathione (GSH) as an electron donor. Eight different isoforms of GPX have been identified, with GPX1 being regarded as the most important [144]. Interestingly, GPX1 is also able to neutralise lipid peroxidation products. GPX1 protein levels and activity were found to be 2.5 to threefold higher in mature oligodendrocytes compared to OPCs [145]. CAT appeared largely inactive in OPCs under oxidative stress, a phenotype that was observed after inhibiting GPX1 as well. This latter finding suggests that GPX1 acts not only as the primary peroxidase but also protects CAT activity [141, 145].

GSH, a tripeptide synthesised from glutamate, cysteine and glycine, is essential for the functioning of GPXs and can also act on its own (non-enzymatically) [146]. GSH can react with O_2_^**·**−^ and hydroxyl radical (OH^•^), forming the oxidised derivative glutathione disulphide (GSSG). GSH levels can be regenerated by reducing GSSG with electrons from NADPH, or by de novo synthesis [143]. The GSH/GSSG ratio is among the most widely used measurements to sense redox alterations. GSH is also able to form adducts with RCS, a reaction that is enhanced by glutathione S-transferases (GST). The GST_∏_ isoenzyme has been detected as a marker for mature oligodendrocytes that is absent in OPCs [147–149]. Moreover, recent studies identified an additional isoenzyme, GSTα4, to do play a role in OPC differentiation and protection against HNE [109]. In line with possessing a threefold lower basal level of GSH compared to astrocytes, OPCs are more vulnerable to GSH depletion [141, 150, 151]. Several oxidative stressors (e.g. H_2_O_2_) are known to cause a profound depletion (~ 40%) of GSH in OPCs already at low concentrations [152, 153]. Other risk factors contributing to the highly vulnerable state of OPCs and (developing) oligodendrocytes are high levels of mitochondrial oxygen consumption, in addition to a greater need than other cells to acquire and retain iron (required for differentiation as well as myelination) [154]. Iron, however, is a catalyst of the Fenton reaction that converts H_2_O_2_ into highly reactive OH^**·**^ [140]. This reaction may be particularly relevant to genomic damage, as iron has been shown to directly interact with the negatively charged phosphate backbone of DNA [155].

Considering their high metabolic load and presence of ROS, it is quite striking that OPCs have such limited antioxidant defence mechanisms. Under oxidative stress conditions, cells can increase their enzymatic and non-enzymatic antioxidant protection, most notably via induction of transcription factor Nrf2, which can bind to antioxidant response elements (ARE) in the promotor regions of genes encoding antioxidant proteins [156, 157]. In MS, Nrf2-driven antioxidant enzyme expression in oligodendrocytes is prominent in actively demyelinating lesions but absent in late-stage active lesions, despite ongoing Nrf2 activation in other cells such as astrocytes [158].

Cells of the oligodendrocyte lineage and in particular OPCs could be considered the Achilles heel to oxidative stress in neurodegenerative disorders, especially due to the immense morphological change in a short time window coupled to limited defence mechanisms against stressors. Whereas high levels of oxidised lipids, protein and DNA are often found in different CNS cells, cerebrospinal fluid or plasma, mostly paralleled by reductions in the antioxidant defence, oligodendrocytes and OPCs show the greatest levels of oxidative damage [89, 159–164]. The apparent mismatch in production and elimination of ROS/RCS in OPCs and oligodendrocytes warrants future research to fully characterise the source and severity of oxidative/carbonyl stress under different pathological conditions.

### Oxidative stress affects OPC differentiation

The question of whether and how oxidative stress impacts oligodendrocyte development is relevant to the study of both CNS development and neurological disease since both require OPC differentiation and (re)myelination. During development, OPCs that do not contact an axon undergo apoptosis, probably contributing to region-specific myelination [165–167]. This axon-dependent OPC survival and differentiation is in part mediated by physiological ROS elevations. Indeed, mimicking neuronal activity in vitro by glutamate-mediated NMDA receptor activation induced OPC differentiation via ROS production, an outcome that was similarly inhibited by NOX blockade [94]. A positive effect of ROS on oligodendrocyte development was also shown by Accetta et al., who observed that H_2_O_2_ treatment could enhance the expression of oligodendrocyte markers (OLIG2, MBP) in MO3-13 cells after 1–4 days, in the absence of any other differentiation-boosting stimuli [83]**.** This effect was dependent on PKC signaling and promoted additional intracellular ROS production by NOX3 and NOX5. Silencing of NOX by siRNAs prevented oligodendrocyte differentiation induced by H_2_O_2_, suggesting that ROS function as intracellular messengers that advance differentiation upon external stimuli.

Yet, it is important to emphasize though that the cellular source, duration and level of ROS dictate the cellular response. Immature oligodendrocytes are considered more vulnerable to several kinds of cytotoxic triggers directly and indirectly related to oxidative stress [89, 141]. In vitro studies clearly show that physiological but sublethal oxidative stress interferes with OPC differentiation without affecting cell viability, and high levels of ROS can induce OPC death. As discussed previously, extensive OPC cell death does not appear as a major feature in neurodegeneration, suggesting that excessive oxidative stress is not attained. ROS exposure during in vitro OPC differentiation reduces mature oligodendrocyte and myelin markers (e.g. GalC, MBP) and alters the expression of cell differentiation regulators [126, 152]. Gene expression of positive differentiation regulators, including SOX10, SHH and HDAC3, was suppressed after a 72 h oxidative stress exposure. Conversely, ID2 and ID4, known inhibitors of differentiation, were increased [152]. Likewise, in vivo data shows that oxidative stress hampers myelination during early development and remyelination during (adult) neurological disease [89, 141]. For example, after white matter injury, ROS blocked a compensatory response in oligodendrocyte regeneration and hampered repair, a phenomenon that was paralleled by functional deficits in working memory similar to neurodegenerative disorders [168]. Compared to ROS, less is known about the direct effects of RCS; however, sublethal concentrations of the lipid peroxidation product HNE were reported to inhibit mitogenic and chemotactic responses to PDGF in undifferentiated OPCs [169]. In addition, reducing mitochondrial HNE levels promotes oligodendrocyte survival and differentiation [109].

It is important to note that most in vitro data supporting the vulnerability of OPCs to ROS used normoxic conditions, potentially meaning altered levels of ROS compared to normal CNS homeostasis [170]. However, collectively, it is hypothesised that elevated ROS/RCS levels in neurodegenerative disorders block the ability of OPCs to differentiate, resulting in a limited CNS (re)myelination capacity. If excessive ROS occurs early in disease, OPC function may start to deteriorate long before the chronic phases that are typically associated with impaired remyelination [171].

## The interplay between ROS/RCS, (epi)genetics, mitochondria, cell signalling and OPC differentiation

**Fig. 3:**
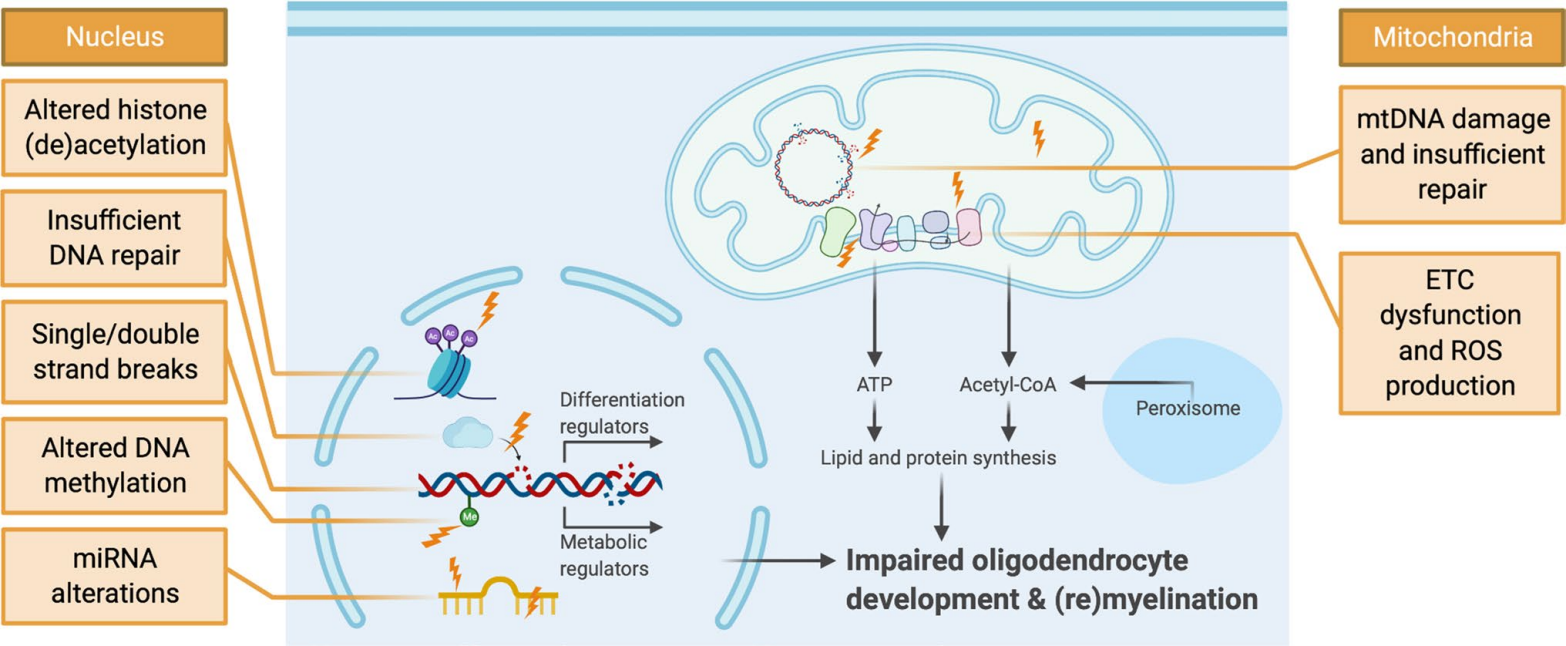
Putative effect of ROS/RCS on OPC (epi)genetics, mitochondria, cell signalling and cell differentiation. Oxidative stress (lightning bolts) affects many cellular compartments. In OPCs, this can lead to inadequate differentiation and impaired (re)myelination capacity. At the nuclear DNA (nDNA) level, oxidative stress may trigger multiple (epi)genetic alterations in OPCs (shown on the left). In addition, oxidative stress-induced changes in metabolism, mitochondrial function and mitochondrial DNA (mtDNA) may also contribute to the observed differentiation block in neurodegeneration. *ETC* electron transport chain. Figure created in BioRender

Despite the evidence that shows a convincing effect of oxidative stress on OPC differentiation, remarkably little research has examined the underlying mechanisms. In this section, we discuss how oxidative/carbonyl stress may interfere with transcriptional and metabolic changes required for OPC differentiation; aiming to guide future research in this area (Fig. 3, Table 1).

**Table 1:** Summary of potential mechanisms involved in impaired OPC differentiation linked to oxidative stress

Mechanism of interest	Description	Effect of oxidative stress	Direct evidence?
nDNA and mtDNA damage	Base modifications (8-oxoG)	Increase	+
	SSB and DSB	Increase	+
DNA repair	Excision repair (NER, BER)	Higher need	+
	Recombinational repair (DSBR)	Higher need	+
	Competing demand: differentiation vs. DNA repair	Increase	±
Epigenetics	HAT and HDAC balance	Altered	+
	Methylation pattern changes	Altered	±
	MiRNA activity and biogenesis	Altered	±
Mitochondria	ETC function	Decrease	±
	ROS generation	Increase	+
Cell signalling	ROS-sensitive signalling in relation to cell growth, metabolism, differentiation and antioxidant defence (e.g. AMPK, MAPK, PGC-1α, Nrf2, mTOR)	Altered	±

+  = direct evidence linking oxidative stress to the mechanism of interest in OPCs, ±  = suggested link but no direct evidence linking oxidative stress to the mechanisms in OPCs yet. *8-oxoG* 8-oxo-7,8-dihydroguanine, *BER* base excision repair, *DSB* double-strand breaks, *DSBR* double-strand break repair, *ETC* electron transport chain, *HAT* histone acetyltransferases, *HDAC* histone deacetylases, *NER* nucleotide excision repair, *OPC* oligodendrocyte precursor cell, *ROS* reactive oxygen species, *SSB* single-strand breaks

### Oxidative stress-induced damage to nDNA and mtDNA in OPCs

Reactive chemicals of oxidative stress can damage all major macromolecules, including DNA. Modifications to DNA can adversely affect the fidelity or operation of many molecular processes such as transcription and replication, and can lead to mutations or genomic instability in OPCs and oligodendrocytes [172]. Similar to neurons, oligodendrocytes and OPCs are known to accumulate DNA damage with age and disease [26]. Aged OPCs, like aged stem cells, show higher levels of oxidative DNA damage than their younger counterparts [33]. OPCs with trauma-induced DNA damage appear to differentiate into oligodendrocytes with a reduced myelination capacity, if they are able to differentiate at all [173]. DNA damage occurs in both nDNA and mtDNA, with the latter being highly vulnerable due to its proximity to the respiratory chain [174]. Below, we touch upon the different mechanisms by which ROS can modify the nuclear and mitochondrial genomes.

Firstly, ROS can attack DNA bases by adding to their double bonds, abstract hydrogen atoms from methyl groups, or attack the sugar residues [175]. Among the DNA bases, guanine (G), which contains a variety of oxidation sites, is the most prone to be attacked by ROS and RCS due to its low reduction potential [176]. The most abundant and most studied product of ROS-induced DNA damage is 8-oxo-7,8-dihydroguanine (8-oxoG), which is mutagenic. It is important to note that in the presence of normal physiological levels of ROS, a certain level of guanine oxidation may be necessary for normal functioning [177]. In MS and other neurodegenerative disorders, increased ROS levels are linked to increased levels of 8-oxoG, in both mtDNA and nDNA [172]. A recent in vivo study, investigating the vulnerability of oligodendroglia after neurotrauma, revealed that OPCs contain more oxidative DNA damage (8-oxoG) than mature oligodendrocytes or axons. HNE levels also appeared to increase most in OPCs after neurotrauma, implicating oxidative/carbonyl stress in the pathological outcome [163].

Secondly, ROS are able to compromise the DNA backbone by introducing primarily single-strand breaks (SSB), but in rare cases, double strand breaks (DSB) [175]. Additionally, when ROS cause SSBs and base modifications in close vicinity of each other (20 bp), these multiple damaged sites, due to their complexity in being resolved, can have profound effects on genome stability and survival. These lesions can, in an attempt to be repaired, be transformed into DSBs when the sugar-phosphate backbone of both strands is broken. Besides causing DSBs indirectly, high levels of ROS can also directly induce frank DSBs [178, 179]. PLP:mtPstI mice, in which DSBs can be timely and reversibly induced in mtDNA from oligodendrocytes, have been used to study the role of DSBs in oligodendropathy [180]. MtDNA encodes several key proteins for the ETC (complexes I, III, IV, V) [181]. MS-like features, including CNS inflammation, demyelination, and axonal injury, were observed in PLP:mtPstI mice following the induction of DSBs in oligodendroglial mtDNA. It was proposed that excessive ROS production amplified these outcomes by causing additional DNA damage and interfering with cell operations [180].

### Oxidative stress and DNA repair in OPCs

During CNS ageing and disease, a complex network of repair pathways functions to detect and resolve DNA damage [182]. Some of the major pathways include excision repair (nucleotide excision repair [NER] and base excision repair [BER]) and recombinational (or double-strand break) repair (DSBR) [183]. Among the different glial cell types, the oligodendrocyte lineage appears to be the least capable of repairing DNA damage. In addition, DNA repair protein levels decrease in adult and aged OPCs compared to neonatal OPCs [34, 184]. NER resolves bulky DNA distortions, including adducts caused by oxidative damage such as cyclopurines. Following recognition, the NER pathways entail the excision of a 25–30 nucleotide segment, leaving behind an undamaged single-stranded DNA gap that a DNA polymerase uses as a template to correctly regenerate double-stranded DNA [185]. BER is involved in the removal and subsequent replacement of primarily non-distorting damaged bases and typically entails just a single-nucleotide re-synthesis event. Glycosylases initiate BER by excising a specific damaged DNA base (for example, 8-oxoguanine glycosylase (OGG1) recognizes 8-oxoG) from the DNA backbone, leaving behind an abasic (or AP) site repair intermediate. The subsequent steps of BER involve single-strand cleavage at the AP site, termini clean-up, repair synthesis and nick ligation to complete the process [186]. Both the nuclear and mitochondrial compartments house DNA repair mechanisms to maintain genome stability, with BER being the primary system in the latter.

Whereas astrocytes were able to fully repair mtDNA damage following a 6 h exposure to menadione, which mimics ETC-induced ROS, oligodendrocytes showed incomplete repair over the same time frame, with decent repair observed during the first 2 h (about 50%) yet minimal repair in the following 4 h (an additional ~ 15%) [184]. The same study showed that menadione exposure leads to more mtDNA breaks in oligodendrocytes than astrocytes, supportive of either increased damage susceptibility or reduced repair capacity. When the concentration of menadione was doubled, more mtDNA breaks occurred in astrocytes as well but they were still able to reach full repair [184]. DNA repair in oligodendrocytes was enhanced by increasing OGG1 activity via transfection of a mitochondrial transport sequence upstream of the OGG1 gene [187], consistent with an overall lower BER capacity.

Recombinational repair (DSBR) has evolved to cope with various forms of DNA DSBs. DSBR mechanisms involve two primary pathways: nonhomologous end joining (NHEJ) and homologous recombination (HR). For the latter, a sister chromatid needs to be available (G2/S phase) to function as a template to permit faithful DSB resolution via the exchange of homologous genetic information. HR, which is tightly integrated with DNA replication to cope with one-ended DSBs that arise during replicative stress, is not vastly utilised in aged OPCs since they are mainly in G0/G1 phase [188]. NHEJ does not require a homologous template and directly anneals the two DNA ends that were formed at a frank (two-ended) DSB. Although NHEJ is the main mechanism in OPCs, it is error-prone as it typically involves the processing of the original sequence by a nuclease or polymerase to facilitate ligation [183, 185, 189, 190]. In many cases, the ability to detect DSBs relies on the MRN complex (composed of MRE11, RAD50 and NBS1). In the oligodendrocyte lineage, CNS-specific inactivation of the *Nbn* gene, which encodes NBS1, leads to hypomyelination via oligodendrocyte apoptosis and a lack of OPC differentiation by dysregulation of transcription regulators (HDACs, MYRF, etc.) [191, 192]. This study illustrates the necessity of DSBR in OPCs.

Based on the limited studies performed thus far, it appears that OPC differentiation can be impeded due to the lack of efficient DNA repair. In addition, future research should explore whether enzymes needed for DNA repair in OPCs are also involved in the cell differentiation program, as these competing interests may hinder differentiation when DNA damage is abundant [172, 193].

### Interference of ROS with epigenetics in OPCs

ROS are known as modulators of the epigenetic machinery. The next sections discuss histone modifications, DNA methylation and miRNAs in the context of ROS and the effect on OPC function and differentiation.

#### Histone modifications, ROS and OPCs

The strongest link between ROS and OPC differentiation, in terms of the epigenetic machinery, has been established for histone modifications. A vast array of changes to histone tails occur, with (de)acetylation of lysine residues being the most prevalent modification [194]. Histone acetyltransferases (HATs) introduce the acetylation, which weakens the interaction of histone proteins with DNA, resulting in a more open chromatin structure. Histone deacetylases (HDACs) exert the opposite effect by removing the acetyl group leading to chromatin compaction and less accessibility for transcription factors. By controlling DNA availability, the interplay between HATs and HDACs influences gene expression profiles [195]. ROS can tip this balance by reducing histone deacetylation, although specific increases in HDAC activity have also been observed [196]. H_2_O_2_ exposure leads to a reduction in overall HDAC levels, as well as HDAC2 inactivation [197]. It is thought that enhanced HDAC activity is dependent on phosphorylation, which can be blocked by ROS such as peroxynitrite [197]. Additionally, RCS (e.g. HNE) are able to cause alkylation of HDACs, inhibiting its function and altering chromatin dynamics [198].

HDAC activity is known to be required for OPC differentiation. Several in vitro and in vivo experiments using (unspecific) HDAC inhibitors have shown a decrease in differentiation, probably linked to failed suppression of inhibitory factors, including ID2, SOX2 and ID4 [54, 199–202]. OPCs exposed to oxidative stress in vitro displayed reduced HDAC expression and activity, resulting in an increased global level of acetylated histone 3 and 4 compared to control [152]. Simultaneous deletion of HDAC1 and 2, which normally repress the Wnt pathway and switch TCF4 to an activator of OPC differentiation, led to severe OPC differentiation impairment [199]. Low HDAC activity in ageing brain also leads to higher expression of differentiation inhibitors SOX2, ID4 and HES5, as well as a downregulation of activator OLIG2 [54]. Using the cuprizone mouse model, it was shown that mimicking brain ageing by pharmacological HDAC inhibition causes defective remyelination [202].

HDACs are also involved in DNA repair. In particular, HDAC1 and 2 can be recruited to DNA lesion sites to deacetylate Lys56 of H3 to facilitate chromatin remodeling. This process is involved in the promotion of NHEJ to repair DSBs. This observation suggests a dual role for HDAC in OPCs, with differentiation and DNA repair competing for its involvement [172]. Altogether, ROS disturb histone (de)acetylation dynamics in OPCs, which is directly linked to the observed differentiation block.

#### DNA methylation, ROS and OPCs

DNA methylation is mainly restricted to the addition of a methyl group (-CH_3_) to the five positions on a cytosine (5mC) base followed by guanine (commonly referred to as a CpG site, of which 60–80% is methylated) [54, 203]. Nearly 10% of CpG sites are found in CpG islands, regions with more than 50% C/G content [54, 204]. Methylation of CpG sites induces gene silencing by blocking the binding of transcription factors and allowing binding of repressor molecules, such as MeCP2 [205]. DNA methylation patterns are controlled by the DNA methyltransferase enzymes DNMT1 (maintenance) and DNMT3a/b (de novo methylation) [206]. DNA demethylation is initiated by hydroxylation of 5mC to 5hmC by ten-eleven translocation (TET) enzymes [54].

During oxidative stress, the formation of the oxidative base lesion 8-oxoG in CpG islands inhibits the binding of any methylation-controlling proteins (DNMT, TET) and disrupts gene expression. In addition, the hydroxylation of 5mC by ROS interferes with epigenetic signaling, since it may mimic demethylation (5hmC) [207]. ROS are also able to increase TET activity and therefore induce additional demethylation and overall hypomethylation [208]. Conversely, ROS can indirectly reduce DNMT activity by reducing the availability of the essential cofactor SAM [208]. However, ROS can also act as a catalyst for DNMTs and promote specific hypermethylation. For instance, O_2_^**·**−^ is capable of deprotonation of C5 on cytosine, increasing base reactivity and allowing more efficient methyl transfer [209]. Moreover, when DSBs occur, H_2_O_2_ exposure can lead to the recruitment of a gene silencing complex that includes DNMT1, resulting in hypermethylation of CpG sites in specific regions of the genome [208].

DNA methylation patterns change during ageing and disease, and can also be observed in response to various exogenous stimuli. While a global reduction of methylation (hypomethylation) is observed in ageing [210], selective hypermethylation has been reported in ageing and neurodegenerative disorders as well. In OPCs, DNA methylation is involved in regulating the proliferative state, but also in controlling alternative splicing and protein synthesis necessary for myelin formation [206]. DNA methylation plays a crucial role in the transition from OPC to oligodendrocyte by regulating its cell cycle exit. The observation that hypomethylation occurs with age also holds true for OPCs. A recent study measuring DNA methylation in OPCs revealed a significant drop in DNA methylation in aged rats, which was consistent with a decrease in DNMT1 expression and activity [211]. The role of DNMT1 in OPC differentiation is further highlighted by the fact that genetic ablation of DNMT1 results in hypomyelination and defective OPC differentiation [212]. Ablation of DNMT3a led to a differentiation block in OPCs and insufficient remyelination after lysolecithin-induced demyelination [213]. Furthermore, OPC differentiation seems to be characterised by a progressive loss of 5hmC signaling, which has received more attention in recent years as being more than just an intermediate during demethylation. ROS are capable of interfering with this pathway by hydroxylation [207, 214]. These observations provide a rationale to further study OPC differentiation in the context of ROS affecting DNA methylation.

#### MicroRNAs, ROS and OPCs

MiRNAs are small non-coding RNAs that make up a family of endogenous gene expression regulators by influencing mRNA translation [54]. This modulatory effect is most often accomplished by means of base-pair complementarity between the miRNA and the 3′-UTR region of the messenger RNA (mRNA) target [215, 216]. The RNA-specific endonuclease Dicer is crucial for the cleavage of pre-miRNA and the formation of the active miRNA species, which can bind to mRNA and accomplish gene silencing [215].

Abnormalities in miRNA biogenesis and function are observed in neurodegenerative disorders (e.g. AD, ALS and PD), and stimulating miRNA biogenesis elicits protective effects in animal models for ALS and PD [217]. Additionally, oxidative stress and the miRNA system appear closely entwined. For example, oxidative stress deregulates both miRNA biogenesis and activity, leading to cellular stress and consequently ROS production. Oxidative stress induction (in silico, in vitro and in vivo) is known to cause a variety of changes to the miRNA system, including both down- and upregulation of specific miRNAs. ROS also affect miRNAs that target oxidative stress-modulating genes and transcription factors [218]. ROS can even induce these changes in miRNA expression through its effect on epigenetic modifications such as the aforementioned ROS-mediated change in DNA methylation and histone modification patterns. Finally, H_2_O_2_ treatment has been shown to decrease the expression of Dicer, which in turn results in decreased miRNA maturation [219].

Several miRNAs have been identified to play a crucial role in OPC differentiation [54]. A microRNAome study indicated three highly induced (10-100x) miRNAs: miR-219, miR-138 and miR-338. The most abundant of the three, miR-219, directly represses the expression of, among others, SOX6 and PDGFαR, which normally inhibit OPC differentiation by promoting proliferation [220]. Therefore, miR-219 is crucial in allowing OPCs to exit the proliferation stage and initiate differentiation. However, in MS, a downregulation of miR-219, as well as miR-338, has been observed in chronic inactive lesions [221]. MiR-27a is also important for cell cycle regulation [222]. A steady-state level of this miRNA appears to be necessary for proper oligodendrocyte development; however, increased levels are associated with impaired OPC differentiation. Interestingly, the negative effect of miR-27a on OPC differentiation was not rescued by co-transfection of miR-219 as assessed by MBP expression. This study indicates that inhibitory cues are powerful and can suppress positive cues [223]. MiR-27a is linked to oxidative stress pathways, since its expression is increased by ROS and miR-27a specifically affects ROS regulating pathways (MAPK, apoptosis, cell survival, etc.) [218, 219]. Furthermore, deletion of Dicer impaired OPC differentiation illustrating that OPCs lacking mature miRNAs undergo some sort of a differentiation block [220].

Collectively, the current evidence indicates that oxidative stress can affect histone modifications, DNA methylation patterns, and miRNA profiles. In doing so, it can interfere with OPC differentiation and contribute to ageing and neurodegenerative disease. The exact mechanisms and links between epigenetic mechanisms and OPC differentiation remain to be elucidated and pose an interesting research field to explore.

### Mitochondrial oxidative damage in OPCs

Insufficient metabolic/mitochondrial adaptations during OPC differentiation can result from the aforementioned (epi)genetic differentiation block. Yet, oxidative stress may also directly impair metabolic processes to ultimately hinder OPC differentiation and (re)myelination.

Mitochondria are redox-sensitive organelles with an essential role in numerous cellular homeostatic processes, while also affecting cell differentiation. Moderate ROS fluctuations are involved in physiological responses, but chronic and/or exaggerated elevations in ROS interfere with mitochondrial function and initiate a vicious cycle of (mitochondrial) ROS production and ROS-inflicted (mitochondrial) damage [224, 225]. Oxidative and carbonyl stress can cause and perpetuate modifications to mitochondrial membrane phospholipids, enzymes and ETC complexes, as well as mtDNA, as discussed above [226–229]. ETC complex I, in particular, is a frequently described target of oxidative modifications implicated in ageing, ischemia–reperfusion, PD and other CNS disorders [230, 231]. Interestingly, when complex I was inhibited in OPCs by a low dose of rotenone that does not compromise cell viability or ATP synthesis, OPC differentiation was blocked in vitro [33, 61]. Differentiating oligodendrocytes were more sensitive to complex I inhibition compared to undifferentiated or already differentiated oligodendrocytes. Complex IV inhibition by sublethal doses of sodium azide briefly prior to or throughout OPC differentiation substantially impaired the formation of complex oligodendrocyte processes [64]. The adverse effect of sodium azide on the mitochondria was intensified in Nrf2-knockdown cells and partly counteracted in Nrf2-hyperactivated (via Keap1 knockdown) cells, implying the importance of antioxidant defence [232]. In addition, homozygous deletion of succinate dehydrogenase subunit D, an important component of both the citric acid cycle and ETC complex II, hindered neuronal and oligodendrocyte (but not astrocyte) differentiation causing brain atrophy [233]. Finally, mice that display abnormalities in ETC oxidative phosphorylation proteins due to eIF2B mutation display defective OPC differentiation with suppressed neurite length in vitro [234].

These lines of evidence clearly suggest an important role for the mitochondrial machinery in OPC differentiation, process formation and myelination. Notably, despite the lack of evidence causally linking ROS/RCS to defects in mitochondrial function and OPC differentiation, it is plausible to hypothesise that oxidative damage to mitochondria elicits such effects.

### Oxidative stress, metabolic signalling and OPC differentiation/myelination

Inhibiting mitochondrial function in OPCs impairs cell growth, differentiation and myelination, and vice versa. Normal mitochondrial biogenesis is controlled by peroxisome proliferator-activated receptor gamma coactivator 1-alpha (PGC-1α), a transcriptional coactivator that interacts with and activates a variety of nuclear transcription factors (e.g. TFAM, PPARs, NRFs, RXRs) [235]. Hereby, PGC-1α also promotes antioxidant defences and cellular lipid metabolism. The regulation and role of PGC-1α in the oligodendrocyte lineage requires further research; however, Jensen et al. suggested that the activation and nuclear translocation of PGC-1α coincide with oligodendrocyte differentiation and is downregulated afterwards [236]. Knockout studies in mice have implicated PGC-1α in myelinogenic programs including oligodendrocyte lipid/cholesterol synthesis and MBP/PLP transcription [236–238]. A deficiency in PGC-1α led to an increase in VLCFAs and a disruption of cholesterol homeostasis in the CNS presumably due to peroxisomal malfunction, whereas enhanced PGC-1α activation accelerated myelin thickening following white matter demyelination in mice [236]. The mitochondrial transcription factor A (TFAM) is a PGC-1α-controlled nuclear-encoded mitochondrial protein that is essential for the maintenance, transcription and replication of mtDNA. Deletion of *Tfam* in Schwann cells caused mitochondrial ETC deficiency, characterised by the preservation of energy levels but a shift in lipid metabolism from fatty acid synthesis toward oxidation. As a result, Viader et al. observed downregulation of lipid synthesis enzymes (e.g. *Srebp1, Fasn*, *Acc2*), depletion of myelin lipid components, chronic demyelination, disturbed glia-axon interaction and axonal degeneration [239, 240]. In addition, peroxisome proliferator-activated receptors (PPARs) have pleiotropic beneficial effects. The α, β and γ isoforms have been shown to boost OPC differentiation under normal conditions and/or preserve OPC differentiation under conditions of oxidative stress or inflammation [85, 241–246]. PPAR effects have been ascribed to their binding to peroxisome proliferator response elements (PPREs) present in the promoter regions of target genes. These include genes involved in lipid/cholesterol metabolism, mitochondria and antioxidant defence, but also in cell plasticity and differentiation programs such as CREB [247, 248].

Interestingly, ROS are well-known activators of PGC-1α signalling. Although underlying signalling pathways in OPCs remain speculative, AMP-activated protein kinase (AMPK) and mitogen-activated protein kinase (MAPK) are potent activators of PGC-1α in response to changes in the cellular redox state and energy levels [249, 250]. The AMPK stimulator metformin has been used to restore the differentiation capacity of aged OPCs, improve remyelination efficiency [33] and protect against demyelination [251]. Although such effects may also be mediated by surrounding CNS cells, Neumann et al. showed that (at least part of) the effect is cell-intrinsic to OPCs and may involve improved mitochondrial function [33]. Likewise, the ROS-induced p38 and ERK MAPK pathways are required to regulate the timing of oligodendrocyte differentiation [252–254] and myelin preservation [255]. Genetic loss-of-function studies strongly suggest a role for ERK1/2 signalling as a positive regulator of myelination both in the central and peripheral nervous system, whilst a gain-of-function in MAPK results in hypermyelination albeit independent of glial cell differentiation [33, 256].

The downstream effects of AMPK and MAPK activation are widespread, but enhancement of mitochondrial biogenesis, antioxidant defence and cellular energy metabolism may explain (part of) the differentiation-enhancing effects of physiological elevations in ROS. However, ageing and various pathologies are characterised by increased oxidative stress, which disturbs, not enhances, mitochondrial biogenesis. Neumann et al. found that aged OPCs from rats exhibit reduced (re)myelination capacity along with elevated p38 MAPK signalling [33]. In addition, H_2_O_2_-induced oligodendrocyte cytotoxicity was accompanied by MAPK activation but prevented by MAPK inhibition [257]. Also, it was hypothesised that the destructive effects of inflammation and oxidative stress on OPCs are mediated by AMPK [258]. The conflicting data on oxidative stress-related pathways draw a more complicated picture and underscore the need for future research to understand how oxidative/carbonyl stress affect different metabolic signals in OPCs. We currently do not know which signals are desired for optimal OPC differentiation, antioxidant defence and myelination. As was suggested for stem cell differentiation [259], the effect of ROS on OPC function is likely dependent on dose, source, and context.

ROS and RCS can disturb metabolic signalling in multiple ways. First, oxidative modifications to important proteins may directly impair signalling responses. However, OPC-specific evidence is currently missing. Proteins involved in antioxidant and mitochondrial signalling that are known to be expressed in OPCs but are vulnerable to oxidative modification include, amongst others, LDL receptor-related protein-1 (LRP1) [260, 261], AMPK [262, 263], and PGC-1α [237, 264]. Second, (over)activation of oxidative stress pathways over long periods may be harmful instead of helpful [250]. This idea was proposed for AMPK activation, which – despite its beneficial effects on mitochondrial adaptations – promotes catabolic over anabolic processes; for example by inhibiting ACC1/2, HMGCR [239, 265, 266], SREBP1/2 [267, 268], and mTORC1 [269, 270]. The aforementioned pathways were found to be involved in the development, growth and myelination capacity of oligodendrocytes. Such observations have led some researchers to suggest that upregulating mTOR in OPCs via AMPK downregulation (instead of AMPK upregulation, e.g. via metformin) may be a therapeutic strategy to boost myelination in neurodegenerative/psychiatric disorders such as schizophrenia [36]. Finally, it also appears that oligodendrocyte Nrf2 and PGC-1α expression is reduced compared to other CNS cell types in MS, suggesting an impaired oxidative stress response [158, 271]. Consequently, oligodendrocytes (and potentially OPCs and developing oligodendrocytes) may lack the physiological adaptations needed to prevent mitochondrial failure, resulting in the inability to protect themselves against oxidative injury.

### Various other effects of oxidative stress

Oxidative and carbonyl stress have been linked to changes in protein (mis)folding, autophagy, endoplasmic reticulum (ER) stress and cellular Ca^2+^ handling. Interestingly, aged OPCs seem more vulnerable to the deleterious consequences of aggregation-prone proteins. Whilst this has mainly been described in neurons from AD, PD and Huntington patients, it also seems to occur in oligodendrocytes [34]. Both the induction and the clearance of protein aggregates may be triggered by oxidative stress. An in-depth review of the aforementioned mechanisms in OPCs is out of the scope of this manuscript.

### Interaction between transcriptional and metabolic events

Above, we discussed separately the effect of oxidative/carbonyl stress on the transcriptional and metabolic regulation of OPC differentiation. However, transcriptional and metabolic events are often tightly interconnected and can reciprocally influence one another. For example, mitochondrial metabolites such as ATP, acetyl-CoA, NAD^+^, α-ketoglutarate and ROS influence (epi)genetic events at the nuclear level (e.g. DNA repair, histone modifications, transcription factors) [272]. Thus, future research investigating ROS/RCS and OPC dysfunction in neurodegeneration should not only keep in mind the involvement of each component but also their interaction.

## Therapeutic approaches to tackle oxidative stress-induced OPC dysfunction in neurodegeneration

It is clear that protecting OPCs under conditions of elevated oxidative/carbonyl stress may hold great therapeutic value in neurodegenerative disorders. To develop fully tailored therapies, a better understanding of the different underlying mechanisms that were covered in this review is warranted. Nevertheless, several interesting therapeutic approaches to tackle oxidative stress-related OPC dysfunction are emerging. Although recently several remyelination-inducing drugs (e.g. anti-LINGO-1, clemastine fumarate) have come to attention, it should be emphasised that so far none of these treatments have reached FDA/EMA approval for induction of remyelination in demyelinating disorders.

By aiming to not only promote OPC survival but also to maintain normal OPC function and (re)myelination, therapies could target either the cause and/or consequences of oxidative and carbonyl stress. Immune-modulatory and anti-inflammatory treatments may protect CNS cells, including OPCs, from excessive oxidative stress [28, 273, 274]. In addition, a variety of specific antioxidant approaches exist. One common goal is to increase cellular GSH, e.g. by administering N-acetylcysteine (NAC) as a source of cysteine (the rate-limiting substrate for GSH synthesis) or by protecting against GSH depletion via increasing alternative antioxidant or carbonyl quenching pathways (e.g. carnosine, aminoguanidine, flavenoids) [275]. NAC has shown beneficial effects in the context of PD, AD, stroke, MDD, schizophrenia, obsessive–compulsive disorder and neuropathic pain [276–278]. Protective effects of NAC have also been demonstrated for oligodendrocytes and OPCs [86, 279–281]. Alternatively, cellular GSH levels, and thereby antioxidant capacity, may be increased by modulating membrane transporters such as the excitatory amino acid transporter 3 (EAAT3). EAAT3 facilitates cysteine transport and is highly expressed in OPCs [282]. Mice overexpressing neuronal EAAT3 contain increased GSH in the cortex, striatum and hippocampus [283]. Whether OPC-specific EAAT3-mediated cysteine transport protects OPCs during oxidative stress is currently under investigation. Stimulating enzymatic antioxidants via the Nrf2/ARE pathway is another promising approach, as recently demonstrated by Lim et al., who reported that Protandim (Nrf2 activator) rescued OPCs from oxidative stress-induced differentiation arrest [126]. Likewise, dimethyl fumarate, another Nrf2 activator, directly impacts oligodendrocytic citric acid cycle intermediates, GSH and lipids, factors that are associated with protection from oxidative stress [284, 285]. Despite the numerous possibilities, many therapeutic options have not yet been explored in OPCs. For example, PGC-1α signalling is an exciting candidate due to its widespread effects on cell metabolism and antioxidant functions. In astrocytes, upregulation of PGC-1α resulted in reduced ROS production and increased resistance to oxidative attack [286]. Several studies have also shown the benefits of PPAR agonists on OPC and oligodendrocyte function under both normal and diseased conditions [85, 241–246]. Activating AMPK signalling via metformin is able to rejuvenate aged OPCs, restoring their differentiation capacity both in vitro and in vivo [33]*.* Finally, Biotin can counter (mitochondrial) oxidative stress, enhance antioxidant function and improve metabolic function (e.g. fatty acid ad cholesterol synthesis) in oligodendrocytes [287], potentially also serving to support OPCs [288]. However, a recent trial reported no efficacy in progressive MS (NCT02936037). As discussed above, the major signalling response in OPCs in different pathological conditions is a topic of hot debate.

Alternatively, several genetic and epigenetic interventions aiming to protect OPCs or boost DNA repair are currently under investigation. These include, for example, DNA damage response (DDR) activators, DNA-binding proteins with epigenetic modifiers, or modulators of HDACs and DNMTs [54, 289]. A variety of non-specific inhibitors against epigenetic enzymes (e.g. TSA for HDAC inhibition, 5-aza for hypomethylation) exist, although they are mostly known in the context of cancer treatment due to their pro-apoptotic effects [290]. When applied to oligodendrocytes, however, these drugs have no beneficial effects, in addition to being non-selective and cytotoxic at high doses [54]. Thus, more selective activation or inhibition of specific enzymes is required. Notably, a recent paper found that activation of HDAC1, which in turn stimulates OGG1 repair activity, offers a therapeutic strategy for the treatment of age- or AD-related cognitive decline and neurodegeneration [291]. In OPCs, specifically, OGG1 enhancement in mitochondria increases mtDNA repair and cell survival after oxidative insults [187, 292]. Other potential approaches to boost DNA repair include the antibacterial agent enoxacin, which is known to enhance Dicer activity. Increased Dicer activity enhances the formation of miRNAs important for the DDR, improving the recruitment of DDR proteins to damaged sites. In HeLa cells, this phenomenon allows more accurate NHEJ and cell survival [289]; however, such approaches remain largely unexplored in the field of OPC research.

## Conclusion

OPC dysfunction is increasingly being linked to many neurodegenerative disorders that are characterised by elevated oxidative and carbonyl stress. Yet, future research with a specific focus on the source and severity of oxidative/carbonyl stress in OPCs under different pathological conditions is warranted. In this review, we have proposed several (epi)genetic and metabolic alterations that could underlie the oxidative stress-induced OPC dysfunction (differentiation block) in neurodegeneration. Deciphering the precise mechanisms of OPC dysfunction during oxidative/carbonyl stress will pave the way for targeted therapeutics with applications in a variety of neurodegenerative disorders.

## Data Availability

Not applicable. Not applicable.
